# Perception of double‐stranded RNA in plant antiviral immunity

**DOI:** 10.1111/mpp.12798

**Published:** 2019-04-03

**Authors:** Annette Niehl, Manfred Heinlein

**Affiliations:** ^1^ Julius Kühn‐Institute, Institute for Epidemiology and Pathogen Diagnostics Messeweg 11‐12 D‐38104 Braunschweig Germany; ^2^ Université de Strasbourg, CNRS, IBMP UPR2357 12 rue du Général Zimmer F‐67000 Strasbourg France

**Keywords:** double‐stranded RNA, pattern‐triggered immunity, receptor recognition, subcellular localization, vesicle transport, virus infection

## Abstract

RNA silencing and antiviral pattern‐triggered immunity (PTI) both rely on recognition of double‐stranded (ds)RNAs as defence‐inducing signals. While dsRNA recognition by dicer‐like proteins during antiviral RNA silencing is thoroughly investigated, the molecular mechanisms involved in dsRNA perception leading to antiviral PTI are just about to be untangled. Parallels to antimicrobial PTI thereby only partially facilitate our view on antiviral PTI. PTI against microbial pathogens involves plasma membrane bound receptors; however, dsRNAs produced during virus infection occur intracellularly. Hence, how dsRNA may be perceived during this immune response is still an open question. In this short review, we describe recent discoveries in PTI signalling upon sensing of microbial patterns and endogenous ‘danger’ molecules with emphasis on immune signalling‐associated subcellular trafficking processes in plants. Based on these studies, we develop different scenarios how dsRNAs could be sensed during antiviral PTI.

## Introduction

Recognition of viral double‐stranded (ds)RNA is a major mechanism in plant antiviral defence via RNA silencing and pattern‐triggered immunity (PTI). dsRNA is a common pattern formed during replication of RNA and DNA viruses (Paudel and Sanfaçon, 2018; Pumplin and Voinnet, 2013; Ramesh *et al*., 2017). Antiviral RNA silencing is initiated by cytosolic dicer‐like proteins, which bind and cleave the dsRNA into short‐interfering RNAs (siRNA) (Guo *et al*., 2019; Yang and Li, 2018). Subsequently, the siRNAs are loaded into ARGONAUTE (AGO)‐containing RNA‐induced silencing complexes (RISC), which then target the same or other viral RNA molecules for sequence‐specific slicing and/or translational repression. PTI has only recently been discovered to act against viruses in plants (Kørner *et al*., 2013; Nicaise and Candresse, 2017; Niehl *et al*., 2016; Yang *et al*., 2010; Zvereva *et al*., 2016). RNA‐based pathogen‐associated molecular patterns (PAMPs) are well‐known inducers of immunity in animals (Chow *et al*., 2018; Hartmann, 2017; Tan *et al*., 2015; Vabret *et al*., 2017), and have been shown to induce immunity upon viral and bacterial infection also in plants (Lee *et al*., 2016; Niehl *et al*., 2016). The mechanism of dsRNA recognition leading to PTI as well as the mode of action against the infecting virus is unknown. However, different plant pattern‐recognition co‐receptor kinases of the SERK (somatic embryogenesis receptor kinase) family, SERK3/BAK1, SERK4/BKK1 and the closely related NIK1 (nuclear shuttle protein [NSP] interacting kinase 1) are involved in the induction of antiviral immune responses upon virus infection in plants (Brustolini *et al*., 2015; Kørner *et al*., 2013; Niehl *et al*., 2016; Yang *et al*., 2010; Zorzatto *et al*., 2015) and viral proteins capable of interfering with these responses were identified (Nicaise and Candresse, 2017; Zvereva *et al*., 2016). While the application of dsRNAs has been shown to induce typical PTI responses dependent on the plasma membrane‐localized pattern‐recognition co‐receptor kinase SERK1 (Niehl *et al*., 2016), the identity of the molecules recognized during SERK3 and SERK4‐mediated antiviral PTI remains unknown. However, dsRNA perception is not affected in *serk3/bak1* mutants (Niehl *et al*., 2016). The defence responses induced by viruses and dsRNA include the production of reactive oxygen species (ROS), induction of hormone signalling, the activation of mitogen‐activated protein kinases, and the induction of defence gene expression (Kørner *et al*., 2013; Nicaise and Candresse, 2017; Niehl *et al*., 2016; Zvereva *et al*., 2016), which are typical responses for antimicrobial PTI (Boller and Felix, 2009). The antiviral immune response mediated by the leucine‐rich repeat receptor‐like kinase (LRR RLK) NIK1 differs from the above‐described model and is specific for infection by begomoviruses (ssDNA viruses). Upon activation of NIK1 during infection, a signalling cascade leading to down‐regulation of translational machinery‐associated genes, and eventually the down‐regulation of viral and host mRNA translation, is induced.

Members of the SERK co‐receptor kinase family are well characterized partners of plasma membrane‐localized pattern‐recognition receptors (PRRs) during antimicrobial PTI (Albrecht *et al*., 2008; Heese *et al*., 2007; Karlova *et al*., 2006; Li, 2010; Li *et al*., 2018b; Roux *et al*., 2011; Santiago *et al*., 2013; Wu *et al*., 2015). However, as viruses are obligate intracellular biotrophs, the question arises how intracellularly produced dsRNAs and proteins come together with these receptors during antiviral PTI. In this short review we describe recent developments in microbe‐associated molecular pattern (MAMP)‐ and danger‐associated molecular pattern (DAMP)‐triggered immune signalling and current insights into immune signalling‐associated subcellular trafficking processes in plants. On the basis of these studies, we develop different scenarios how dsRNAs could be sensed during antiviral PTI.

### Extracellular perception of dsRNA

**Figure 1:**
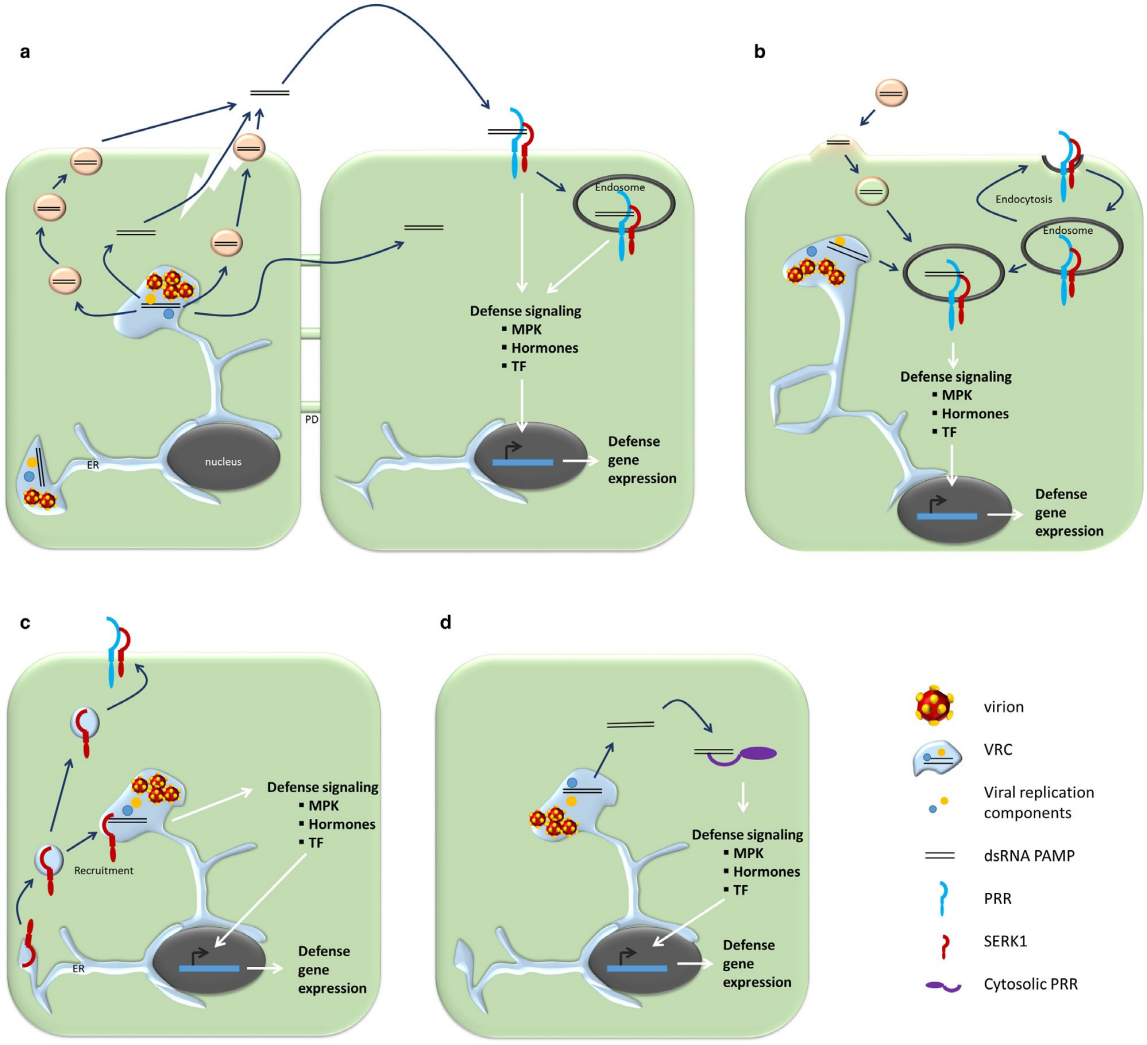
Model illustrating different scenarios for the perception of dsRNA during dsRNA‐mediated antiviral innate immunity (a) dsRNA produced during virus replication may be transported between cells through plasmodesmata (PD) or reach the apoplast through active vesicle‐mediated transport or from damaged cells. Extracellular dsRNA may be perceived by the same or neighbouring cells via plasma membrane (PM)‐localized pattern‐recognition receptors (PRR) and the co‐receptor kinase SERK1. Subsequently, the ligand‐receptor complex may undergo endocytosis, as common for ligand‐PRR complexes during plant immunity. (b) The receptor complex may cycle between the PM and endosomes in the absence of the dsRNA ligand. In this case, recognition may take place after fusion processes between endosomal‐localized receptors and dsRNAs internalized into vesicles or after membrane fusion processes of endosomal‐localized receptors and viral replication compartments (VRCs). (c) Viral replication‐associated processes may lead to the recruitment of the co‐receptor kinase SERK1 and other membrane‐localized or cytosolic PRRs (not shown) to VRCs, where they could participate in the recognition of dsRNA produced during virus replication. (d) dsRNAs accumulating during virus replication may be directly sensed by cytosolic PRRs. In all scenarios, dsRNA recognition results in the activation of defence signalling and PTI. White arrows indicate signalling events, dark arrows indicate transport processes. dsRNA, double‐stranded RNA; ER, endoplasmic reticumum; MPK, mitogen‐activated protein kinase; PAMP, pathogen‐associated molecular pattern; PRR, pattern recognition receptor; SERK1, somatic embryogenesis receptor kinase1; TF, transcription factor.

The simplest assumption for the induction of PTI by dsRNA in plants is a mechanism analogous to the perception of typical microbial PAMPs such as flagellin/flg22 or fungal chitin. During the perception of extracellular microbial PAMPs, PRRs at the plasma membrane specifically bind these PAMPs in concert with a pattern‐recognition co‐receptor. Binding of the PAMP then induces receptor phosphorylation, the recruitment of signalling components, and the induction of downstream signalling cascades (Alhoraibi *et al*., 2018; Boller and Felix, 2009). For activation of antiviral PTI, the viral dsRNA may be released into the apoplast during infection. This could occur either by passive release from damaged neighbouring cells, or as a result of an active transport out of the cell (Fig. 1a). Extracellular dsRNA may then be specifically perceived by a plasma membrane‐associated PRR, which may form an active receptor complex with SERK1 and induce PTI signalling in a way similar to the perception of other previously characterized PAMPs (Fig. 1a). A precedent for the involvement of a release of signalling molecules into the apoplast by cells for recognition by PRRs at the plasma membrane is exemplified by the perception of endogenous danger‐peptides. Here, release of intracellular peptides during wounding and perception through surface receptors at neighbouring, intact cells induces immune signalling by signalling cascades analogous to the perception of microbial PAMPs (Albert, 2013; Bartels and Boller, 2015; De Lorenzo *et al*., 2018).

Evidence for the transport of functional RNA molecules across plant membranes has been provided by a cross‐kingdom RNAi study, where long dsRNAs sprayed onto plants were able to induce RNAi against fungal genes important for *Fusarium graminearum* ergosterol biosynthesis and to inhibit fungal growth in tissues distant from the spray site, suggesting that exogenously applied dsRNA is taken up and transported inside plants, and capable of reaching fungus in systemic plant tissues (Koch *et al*., 2016). Experimental evidence for such interorganismal RNA transport was also provided in a study investigating the effect of cotton miRNAs on fungal gene expression (Zhang *et al*., 2016b). Plant miRNAs were detected inside fungal hyphae and shown to down‐regulate fungal target gene levels, thus demonstrating transport of miRNAs from the plant to the fungus. Export of dsRNAs from plant cells may occur in form of extracellular vesicles. The presence of functional RNAs in extracellular vesicles is a well‐ known feature in mammals (Conigliaro *et al*., 2017) and has only recently been discovered in Arabidopsis (Cai *et al*., 2018a, 2018b, 2018a, 2018b; Zhao *et al*., 2018). The findings imply the existence of a functional exosome pathway that delivers sRNAs into the extracellular space and into fungal pathogens for the silencing of fungal genes critical for pathogenicity during *Botrytis cinerea* infection (Cai *et al*., 2018b). Moreover, although plant viruses are transported between cells through plasmodesmata, recent evidence indicates that an alternative viral trafficking pathway involving apoplastic vesicles might exist. Using high‐end microscopy, it was shown that vesicles containing viral RNA occur outside the cells and in xylem vessels during *Turnip mosaic virus* and *Potato virus X* infection (Wan *et al*., 2015; Wan and Laliberté, 2015). Although the authors suggest that the virus may move through pit membranes from phloem into immature xylem for replication before cell death occurs, the above findings could suggest the possibility that the viral RNA may enter xylem by an exosomal pathway.

### Endomembrane‐associated intracellular perception of dsRNA

During virus infection in animals, dendritic cells and macrophages internalize virus particles and/or extracellular vesicles containing viral RNA. The RNAs are then released into endosomes, where they are recognized by Toll‐like receptors to induce immune responses (Baglio *et al*., 2016; Brencicova and Diebold, 2013; Dreux *et al*., 2012; Kouwaki *et al*., 2016, 2017, 2016, 2017; Matsumoto *et al*., 2014; Okamoto *et al*., 2014). In analogy to the mammalian system, it is known that bacterial PAMP receptor‐complexes in plants re‐localize from the plasma membrane to endosomes (Avila *et al*., 2015; Beck *et al*., 2012; Frescatada‐Rosa *et al*., 2015; Russinova *et al*., 2004) and that plant receptor proteins can signal from endosomes during immunity and development (Geldner *et al*., 2007; Irani *et al*., 2012; Mbengue *et al*., 2016; Sharfman *et al*., 2011). Thus, it is conceivable that the putative membrane‐localized PRR receptors for dsRNA may cycle between the plasma membrane and endosomal compartments, and that recognition may take place during membrane fusion events between receptor‐containing endosomes and viral RNA‐containing vesicles or viral replication compartments (VRCs, Fig. 1b). The latter may appear likely given that plant viruses generally replicate their genomes in close association with endomembrane compartments (Jin *et al*., 2018; Laliberté and Sanfaçon, 2010; Verchot, 2011). In the case of the model plant virus *Tobacco mosaic virus* (TMV), for example, replication takes place in viral replication complexes embedded in modified ER (Heinlein *et al*., 1998; Reichel and Beachy, 1998). TMV replication complexes associate with the ER and microtubules at cortical sites that may present direct contact with the plasma membrane (PM) and PM‐located proteins (Niehl *et al*., 2013; Peña and Heinlein, 2013; Pitzalis and Heinlein, 2018), thus presenting a possibility for interaction between intracellularly produced viral dsRNA replication intermediates and membrane‐localized receptors. Typically, plant virus replication on membranes induces strong membrane modifications and is associated with interference of virus infection with lipid metabolism and membrane targeting and transport (Altan‐Bonnet, 2017; De Castro *et al.*, 2013; Nagy, 2016; Nagy and Pogany, 2012; Zhang *et al*., 2016a, 2018). Infection by diverse plant viruses has been shown to imply membrane fusion processes (Garcia Cabanillas *et al*., 2018; Huang *et al*., 2015; Wei *et al*., 2013) as well as conventional and unconventional protein transport routes (Diaz *et al*., 2015; Grangeon *et al*., 2012; Kovalev *et al*., 2016; Movahed *et al*., 2019; Ribeiro *et al*., 2008; Wang *et al*., 2011; Wei and Wang, 2008).

dsRNA recognition by PRRs and the induction of antiviral PTI at the membrane‐associated VRCs could also occur through recruitment/mistargeting of the membrane‐localized receptors during infection (Fig. 1c). Indeed, many host factors are recruited to VRCs during virus infection (Nagy and Pogany, 2012). Tombusvirus replication, for instance, induces a replication compartment consisting of aggregated peroxysomes, endoplasmic reticulum and early endosomal proteins and membranes (Nagy, 2016). The tombusvirus p33 replication protein co‐opts host factors involved in membrane fusion for virus replication (Sasvari *et al*., 2018). Also the *Turnip mosaic virus* (TuMV) 6K2 protein has recently been shown to interact with and relocate a host protein involved in ER fusion for efficient replication and movement (Movahed *et al*., 2019). With respect to folding and quality control of immune receptors, it was shown that the Hsp70/Hsp90 organizing co‐chaperone Hop/Sti1, which is involved in the maturation of PRRs, presumably also assists maturation of PRRs involved perception of *Potato virus Y* (PVY) and becomes recruited into VRCs during infection (Lamm *et al*., 2017). Moreover, ER‐localized chaperones are important for the expression of a plasma membrane‐localized receptor‐like kinase required for the N‐mediated hypersensitive response leading to resistance against TMV through programmed cell death and systemic acquired resistance (Caplan *et al*., 2009). Thus, recruitment of host factors to VRCs may directly or indirectly affect virus recognition by receptor proteins. Despite of the fact that the interaction of the viral protein with the host protein folding machinery was shown to be critical for evasion of recognition in these studies, it is conceivable that the mistargeting of host factors during membrane rearrangement in virus replication may also lead to recognition of viral dsRNA replication intermediates by the host.

### Intracellular recognition of dsRNA

Apart from membrane‐associated dsRNA recognition events, plants may sense dsRNA PAMPs also by PTI receptors in the cytoplasm. In animal cells, cytosolic RIG‐I (retinoic acid‐inducible gene‐1)‐like receptors (RLR) recognize and bind viral RNA and dsRNA (Hornung, 2014a, 2014b). Interestingly, RLR proteins share a conserved Duplex RNA‐activated ATPase (DRA) domain important in viral RNA sensing with Dicer proteins, which are key players in RNA silencing (Paro *et al*., 2015), and structural studies indicate that Dicers and RLRs undergo similar conformational changes upon dsRNA binding. In this context it appears interesting that the RLR LGP2 (Laboratory of Genetics and Physiology 2) binds to and inhibits Dicer activity *in vitro* and in cells (van der Veen *et al*., 2018), while it acts as a cofactor to the RLR MDA5 (melanoma differentiation‐associated factor 5) and supports activation of interferon‐mediated antiviral immunity by long dsRNA (Bruns and Horvath, 2015). Thus, due to their sequence similarity with animal RLRs, dicer‐like proteins (DCLs) are obvious candidates for plant cytosolic dsRNA receptors. However, PTI responses were not impaired in *dcl* mutants (Niehl *et al*., 2016), indicating that dsRNA‐mediated antiviral silencing and dsRNA‐mediated antiviral PTI are distinct plant defence pathways. Nevertheless, the two pathways may be functionally linked. *rdr6* mutants impaired in the production of long dsRNAs as templates for secondary siRNA synthesis show constitutively activated PTI. Thus, the induction for RDR6 activity during viral infection may play a role in down‐regulating PTI‐related defence genes (Boccara *et al*., 2014). Indeed, numerous nucleotide‐binding and leucine‐rich repeat (NB‐LRR) disease resistance genes involved in PTI and effector‐triggered immunity (ETI) are post‐transcriptionally controlled by conversion of their transcripts to dsRNA by RDR6 and subsequent cleavage into sRNAs by DCL enzymes (Boccara *et al*., 2014; Li *et al*., 2012; Shivaprasad *et al*., 2012; Yi and Richards, 2007). It thus appears that the induction of antiviral silencing and the production of secondary antiviral siRNAs by RDR6 could involve the concomitant down‐regulation of PTI and ETI genes and that PTI and ETI may represent a safeguard strategy in case that RNA silencing mechanisms fail to be effective to counter virus infection. Whether such a safeguard mechanism may function also in the opposite way remains to be investigated. It is interesting that certain viral proteins identified as suppressors of RNA silencing also act as suppressors of PTI (Love *et al*., 2012; Zvereva *et al*., 2016). This again supports the interaction between the two defence pathways and is consistent with co‐evolution of plant antiviral defence and viral counter‐defence mechanisms.

**Figure 2:**
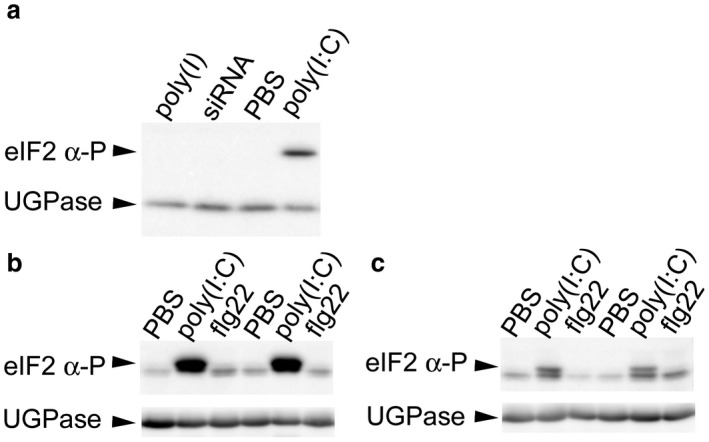
dsRNA treatment induces phosphorylation of eIF2 alpha in Arabidopsis leaves (a) Treatment of Arabidopsis leaf discs with 500 ng/μL poly(I:C), but not with 500 ng/μL poly(I), or with 2 μM siRNAs or PBS induced eiF2 alpha phosphorylation. (b) and (c) Treatment of Arabidopsis leaf discs with 500 ng/μL poly(I:C), but not the treatment with PBS or 1 µM flg22 induced eiF2 alpha phosphorylation. Leaf discs were incubated in 24 well plates in water overnight and then incubated for 24 h (a) and (b) or for 45 min (c) with the substances. Following induction, leaf discs were frozen in liquid N_2_, proteins extracted and used for western blot analysis using antibodies specific for phosphorylated eIF2 alpha and UGPase and HRP‐labelled goat anti‐rabbit secondary antibodies. Blots were developed by luminescence detection.

Another intracellular antiviral defence mechanism in animal cells operates through binding of viral dsRNAs by the eukaryotic initiation factor eIF2‐alpha kinase PKR, which induces a shutdown of protein translation as part of an antiviral defence response (Dalet *et al*., 2015; Dar *et al*., 2005; Dey *et al*., 2005). eIF2 alpha phosphorylation by PKR leads to compromised availability of loaded Met‐tRNAs, hence leading to an overall reduction of protein translation (Dzananovic *et al*., 2018; Sonenberg and Hinnebusch, 2009). As already mentioned, a shutdown of protein synthesis is also part of the defence pathway against begomovirues, which involves activation of the SERK‐related receptor protein kinase NIK1 (Zorzatto *et al*., 2015). Upon infection, NIK1 triggers a signalling cascade leading to a shutdown of ribosomal gene expression and, in consequence, inhibited viral and host protein expression. This reaction is counteracted by the virus‐encoded virulence factor NSP, which shuttles the replicated viral ssDNA genome from the nucleus to the cytoplasm and also binds to NIK1 to inhibit its antiviral signalling activity (Calil and Fontes, 2017; Gouveia *et al*., 2016; Nicaise, 2015; Zorzatto *et al*., 2015). It remains to be seen whether dsRNA perception and antiviral PTI are entangled with a pathway leading to the shutdown of protein synthesis in plants. Strikingly, treatment with the dsRNA analogue poly(I:C) induced phosphorylation of Arabidopsis eIF2 alpha, while treatment with the single‐stranded RNA mimic poly(I) and treatment with siRNA did not (Fig. 2a). Infection with *Turnip crinkle virus, Turnip yellow mosaic virus* or *Potato virus X*, however, does not appear to induce eIF2 alpha phosphorylation in plants (Meteignier *et al*., 2016; Zhang *et al*., 2008). It remains to be seen whether other viruses induce eIF2 alpha phosphorylation in plants or whether virus‐encoded inhibitors interfere with this plant response. eiF2 alpha phosphorylation did also not occur upon application of the bacterial PAMP flg22 (Fig. 2b,c). Nevertheless, eIF2 alpha phosphorylation has been shown to take place in response to *Pseudomonas syringae* infection and upon infection by *Bemisia tabaci* (Li *et al*., 2018a; Pajerowska‐Mukhtar *et al*., 2012) as well as in response to treatment with plant hormones involved in pathogen defence and the priming agent beta‐aminobutyric acid (Lageix *et al*., 2008, Li *et al*., 2018a, Liu *et al*., 2015, Luna *et al*., 2014, Wang *et al*., 2017). Thus, additional studies are needed to clarify the specific function of eIF2 alpha phosphorylation upon biotic stress and to further define the conditions under which it is induced. Stress‐induced phosphorylation of eIF2 alpha triggers the formation of cytosolic, subcellular RNA granules called stress granules, where preassembled ribosomal pre‐initiation complexes are stored (Mäkinen *et al*., 2017). An interesting question to be resolved in the future is whether cytosolic RNA granule‐associated RNA binding proteins involved in the recognition and degradation of viral RNAs (Mäkinen *et al*., 2017) may prove to be involved in dsRNA perception during dsRNA‐mediated antiviral PTI.
